# Waste Glass Upcycling Supported by Alkali Activation: An Overview

**DOI:** 10.3390/ma17092169

**Published:** 2024-05-06

**Authors:** Muhammad Jamshaid Zafar, Hamada Elsayed, Enrico Bernardo

**Affiliations:** Department of Industrial Engineering, University of Padova, Via Marzolo 9, 35131 Padova, Italy; muhammadjamshaid.zafar@studenti.unipd.it

**Keywords:** alkali-activated materials, waste glass, porous geopolymers, sustainable building materials

## Abstract

Alkali-activated materials are gaining much interest due to their outstanding performance, including their great resistance to chemical corrosion, good thermal characteristics, and ability to valorise industrial waste materials. Reusing waste glasses in creating alkali-activated materials appears to be a viable option for more effective solid waste utilisation and lower-cost products. However, very little research has been conducted on the suitability of waste glass as a prime precursor for alkali activation. This study examines the reuse of seven different types of waste glasses in the creation of geopolymeric and cementitious concretes as sustainable building materials, focusing in particular on how using waste glasses as the raw material in alkali-activated materials affects the durability, microstructures, hydration products, and fresh and hardened properties in comparison with using traditional raw materials. The impacts of several vital parameters, including the employment of a chemical activator, gel formation, post-fabrication curing procedures, and the distribution of source materials, are carefully considered. This review will offer insight into an in-depth understanding of the manufacturing and performance in promising applications of alkali-activated waste glass in light of future uses. The current study aims to provide a contemporary review of the chemical and structural properties of glasses and the state of research on the utilisation of waste glasses in the creation of alkali-activated materials.

## 1. Introduction

The emergence of alkali-activated material (AAMs) as a sustainable substitute for Portland cement (PC) encourages the usage of various types of silicate and aluminosilicate sources for production [1,2,3]. The most common precursors in alkali-activated materials are metakaolin, fly ash, and slag [4,5,6]. However, widely used materials like fly ash and powdered, granulated furnace slag are not always accessible in certain areas, which raises production costs and adversely affects the environment during transportation. The growing awareness of environmental issues in nations like Italy has led to the closure of coal power facilities, negatively impacting the supply of fly ash that can be used to make AAMs [7,8,9]. Thus, an area of particular interest is the potential use of supplementary aluminosilicate materials to cope with possible shortages of fly ash and metakaolin.

Among supplementary silicate and aluminosilicate materials, glass deserves particular attention. Glass accounts for a sizeable portion of overall solid wastes that is accessible every year around the globe. In general, glass is a highly chemically stable substance that can be recycled infinitely without degradation of its molecular structure (typically observed with thermoplastic polymers, which undergo chain cracking upon remelting, with a reduction in molecular weight). Therefore, waste glass management and collection are becoming increasingly prevalent components of environmental planning in developed regions [10,11]. According to recent data, over 13.5 million tons of glass containers are recycled annually in Europe, at a recycling rate of over 78% [12]. The recovered glass, however, must adhere to several requirements for reuse in new glass articles: it must be in a ”closed-loop recycling” condition (meaning that the material is from a discarded article, of a first generation, or used as feedstock for a similar article of a second generation). Industrial and urban waste glass is often gathered and sorted according to typology. However, the variety of glass formulations and their mixing with other materials (polymeric, metallic, ceramic impurities) make a complete ”closed-loop recycling” unfeasible. As a result, almost 30% of discarded glass objects lead to waste material, necessitating the search for other valorisation pathways [13,14].

Using waste glass as a precursor for making AAMs is an innovative way to recycle glass powders into building materials. In alkali activation, precursors dissolve in the activating medium, —consisting of a concentrated aqueous solution of alkali hydroxides or alkali silicates—where the degradation products condense and polymerise, leading to products with variable molecular structures. A three-dimensional, continuous, highly interconnected structure is available in the subset of AAMs known as ”geopolymers” [15,16]. These products feature a ”zeolite-like” network, i.e., they feature a specific connectivity between structural units, finding analogies in the same zeolites, in feldspar minerals, or in alkali aluminosilicate glasses [17]. Bridging oxygens connect both SiO_4_ and AlO_4_ tetrahedral units, the latter being stabilised by alkali ions in the surroundings. Variants may also feature BO_4_ and FeO_4_ units [18]. When using glass as a precursor, the achievement of a real ”zeolite-like” network is conditioned by the availability of the above-mentioned units, which is based on the chemical formulation. It should be noted that some authors, e.g., [19], associate the term ”geopolymer” with a strict balance among constituents, expressed, as an example, by the formula Na_2_O·Al_2_O_3_·4SiO_2_·nH_2_O (for Na-based activation), which also evidences that the products are still ”hydrated” (in this case, that the Al and Si ions are not completely surrounded by bridging oxygens but also bound to –OH groups).

To create alkali-activated mortars from three types of recycled glass, namely hollow, flat, and windshield panes, Idir et al. used KOH as an alkali activator at different concentrations and varying curing duration. The results indicated that, for all three types of recycled glass, the best conditions for synthesis were three mol/l KOH and seven days of curing at 60 °C [20]. Also, a study on ground blast-furnace slag-based alkali-activated mortars containing waste glass was carried out by Zhang et al. In creating alkali-activated mortars, waste glass cullet was used in place of natural aggregates, and glass powder was used in place of ground blast-furnace slag. As a result, mortars with a compressive strength of 40 MPa were produced by replacing more than 70% (by mass) of the slag with glass powder [21]. Most recently, using the weak-alkali activation method, Ramteke et al. obtained highly porous glass foams from waste glass with specific chemical compositions, such as opal glass and glass fibre waste. Opal-glass-based foams demonstrated an excellent uniform open porosity. Time and energy usage were minimised in both cases [22]. The studies mentioned above have demonstrated that using glass waste in producing building materials such as cementitious materials and geopolymers will significantly reduce the ecological footprint and cost of the materials. Glass powder can be used as a precursor in producing AAMs due to its high silica concentration; the binding of adjacent particles, however, is not strictly dependent on the formation of a truly zeolite-like gel [23].

There have been few studies on the use of waste glass in AAM production in comparison with traditional precursors such as fly ash and slag. This detailed review is conducted to explore the optimal conditions for producing durable and economical alkali-activated material from recycled waste glass. This study set out to discover how intrinsic factors, such as the type and amount of the alkaline activator and curing conditions, glass composition, the microstructure of the reaction products, and the types of gels formed, affect the final products. The discussion in this article is a helpful resource for the stakeholders interested in using alternate precursors for producing AAMs. Additionally, this review study will encourage additional research on waste glass, its development, and its applications in AAMs.

## 2. Overview of Waste Glass Used in Alkali Activation

**Figure 1:**
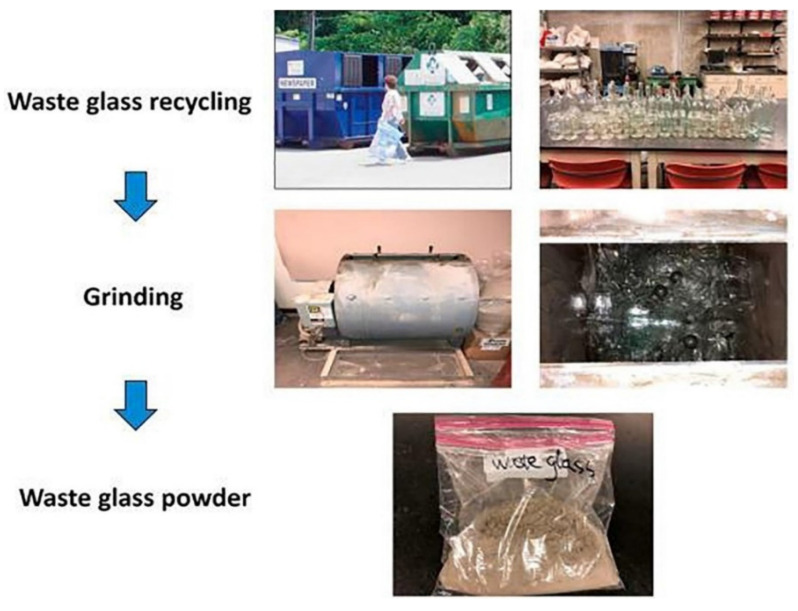
Simplified glass powder production process [25].

Waste glass can be categorised based on its chemical structure and the additives used during production. Table 1 lists the different types of glasses, their applications, and their chemical composition. Some examples of such glass are soda-lime glasses, aluminosilicate glasses, lead glasses, and borosilicate glasses. More than 82% of all glass trash is soda-lime glass, most of which originates from bottles and other containers in the urban waste stream [24]. Glass from fluorescent light bulbs and sheet glass, both of which are of the soda-lime type, have higher impurity levels than container glass trash, which has higher silica and calcium oxide contents. Instead of letting heavy metals in glass trash leak into landfills, including them in alkali-activated material is considered an efficient stabilizing technique [25]. Figure 1 illustrates the conversion process of glass waste into glass powder.

### 2.1. Soda-Lime Glasses

Soda-lime glass is the most common glass produced using the sodium–calcium silicate technique. The majority of commercial glass is soda-lime glass. It can be recycled easily and is comparatively less expensive. This glass typically has 70–75% silicon dioxide, 11–16% sodium oxide, 10–15% calcium oxide, 1–4% magnesium oxide, and 1.2–2.0% aluminium oxide. To tailor its characteristics and suit a variety of uses, additional reagents may be mixed in at a low concentration. Sodium oxide, sometimes known as soda, is the primary additive in this form of glass, apart from silica [26]. Glass containers, bottles, and windows are all typical applications for soda-lime glass, which is mass-produced. It may be used as window glass due to its low melting point and high light transmission. Its smooth, nonreactive surface is perfect for food and drink storage containers. Soda-lime–silicate glass waste, often derived from containers and flat glass, is the most abundant glass waste [27].

Waste glass can be recycled in many ways, but producing alkali-activated materials is the most promising and eco-friendly method to recycle waste glass. Recent research suggests that adding waste glass (especially soda lime) to either Portland or non-Portland binders improves their quality when fresh and after hardening. Ordinary Portland cement (OPC) and alkali-activated materials (AAMs) may benefit from glass waste. It serves various purposes in various binders, such as an aggregate, a precursor, and an activator [28,29].

**Figure 2:**
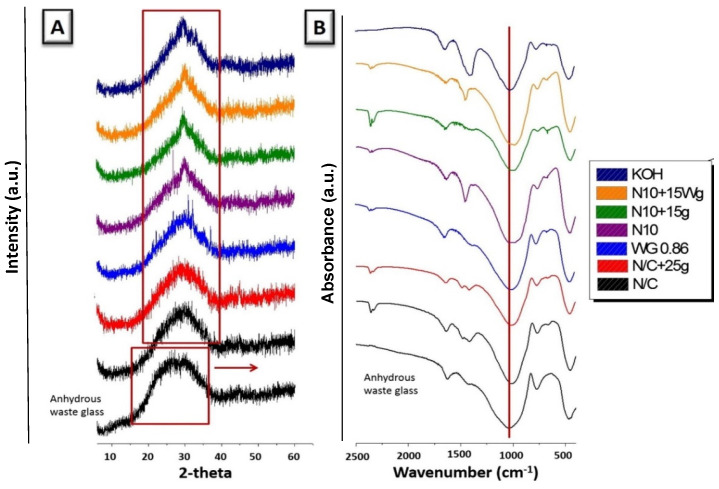
Unhydrated waste glass and activated waste glass pastes: (**A**) XRD patterns; (**B**) FTIR spectra. Varying amounts of the alkaline activators NaOH, NaOH/Na_2_CO_3_, sodium silicate hydrate, and KOH were used to prepare waste glass pastes (modified) [33].

Recent studies have shown that using soda-lime waste glass as a silica supply negates the need for a water-glass solution as a chemical activator [30]. The addition of waste glass as a precursor shows better dissolution characteristics in alkaline media, and the strength of the hardening binders is also gradually improved [31]. According to Cyr et al., soda-lime glass waste can be converted into inorganic polymers in relatively weak alkaline solutions (3 M KOH). The hardening of suspensions relied on the creation of “tobermorite-like gels” or gels based on calcium silicate-hydrated compounds (C-S-H) to create layered structures, because Al_2_O_3_ was almost absent in soda-lime glass [20,32]. The waste soda-lime glass was amorphous, with a low-range structural order in the X-ray diffraction analysis (XRD) for the waste glass and the pastes made with the activators. The corresponding XRD trace showed an amorphous hump, with no reflections attributed to any discernible crystalline component (Figure 2). The XRD results demonstrated that the reaction products in the pastes with each activator kept an amorphous form [31,33,34].

### 2.2. Borosilicate Glasses

Borosilicate glass is a kind of glass that uses silica and boron oxide as its primary glass-forming ingredients. Unlike other common glasses, borosilicate glasses are very resistant to thermal shock due to their extremely low coefficients of thermal expansion. Silica (70–80%) and boric oxide (7–13%) are the major components of borosilicate glass, while the alkalis (sodium and potassium oxides) and aluminium oxide (1%) round out the formula [35]. Due to its low alkali content, this glass offers excellent chemical durability and thermal shock resistance. For this reason, it finds extensive use in the manufacture of laboratory equipment, ampoules, and other pharmaceutical containers. Boron is used to produce borosilicate glass in addition to the more common glassmaking ingredients like quartz, sodium carbonate, and calcium carbonate, which modify the glass characteristics for a specific use. The same role is played by boron in glassmaking as it does in ceramics at low temperatures [36].

Pharmaceutical glass is a borosilicate with a unique chemical composition, making recycling challenging. Fine powders of boro-aluminosilicate glass are partially dissolved in a moderately basic solution of sodium and potassium hydroxides, known as alkali activation, an environmentally friendly method for recycling this type of glass. Although the lack of CaO in alkali-activated pharmaceutical glass prevents the creation of C-S-H compounds, alkali-activated suspensions may still be gelled and used to produce glass foams and other porous structures.

**Figure 3:**
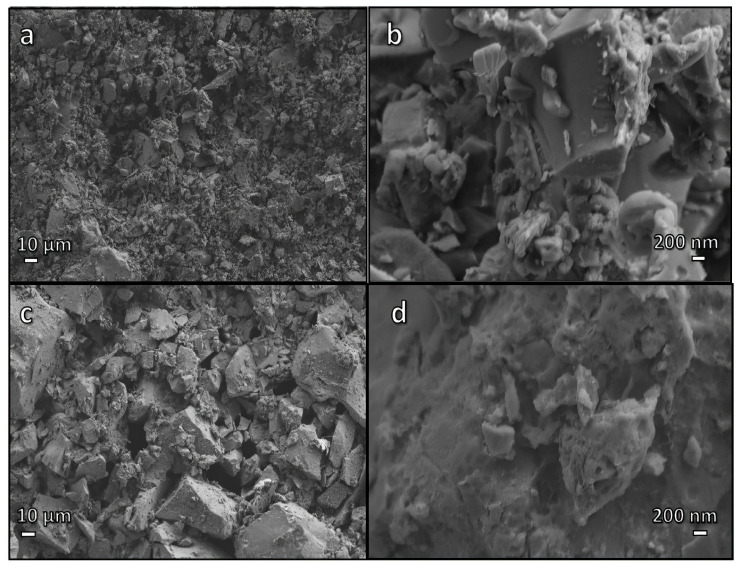
Sample micrographs at low and high magnifications after cold consolidation: (**a**,**b**) a simply hardened suspension; (**c**,**d**) after a boiling test [39].

According to nuclear magnetic resonance (NMR) measurements, the activation of pharmaceutical glass waste with NaOH/KOH produced amorphous materials with a predominance of AlO_4_ and BO_4_ units. Traces of hydrated sodium carbonate were present in foamed solutions, and a freshly produced amorphous gel followed this phase. Drying activated glass suspensions without creating foam produced sodium- and potassium-based hydrated carbonates. Hydrated carbonate phases did not cause the hardening of the activated glass suspensions. The overlapping contributions of hydrated carbonates embedded in an amorphous gel matrix near the surface of glass particles cause the observed gelation (Figure 3a,b). Alkali ions in foams likely did more than stabilise the AlO_4_ and BO_4_ units in the gel; they also likely encouraged the construction of a highly depolymerised network, inhibiting the condensation of silanol groups and the development of robust Si-O bridges. Glass particles were most likely “glued” together by the condensation of silanol groups since alkali ions entered the gel significantly less often in denser bodies [37,38,39].

Recently, Mehta et al. used waste glass from the pharmaceutical industry to create very homogeneous cellular architectures. First, fine particles are activated by being mixed with weakly alkaline solutions (2.5 M NaOH/KOH), and then the partly gelified suspensions are mechanically stirred while being doused with surfactant. The next step involves a fire between 550 and 650 degrees Celsius, where the material is dried and hardened. Thermal decomposition of the gel phase, as opposed to the viscous-flow sintering of glass, enables the stabilisation of cellular structures at temperatures as low as the glass transition temperature of the employed glass [39].

### 2.3. Aluminosilicate Glasses

Most aluminosilicate glasses have a composition of 52–58% silicon dioxide, 15–25% aluminium oxide, and 4–18% calcium oxide and are typically made using a ternary method. This glass is utilised in thermometers, combustion tubes, cookware, halogen lights, furnaces, and fibreglass insulation due to its low thermal expansion and softening temperature. Aluminosilicate glasses may achieve high surface strength by adding more alkalis. Smartphone displays and airplane windscreens use this sort of glass, and other potential uses (such as in automobiles) are on the horizon. Most glass–ceramics developed for consumer applications start as alkali or alkaline-earth aluminosilicate systems [40,41]. Large quantities of aluminosilicate glass such as glass fibre are manufactured each year commercially for use in various contexts. Unfortunately, millions of tonnes of unwanted fibreglass are produced as a byproduct of this procedure. The chemical composition of this waste fibreglass mainly consists of vitreous silica, alumina, and calcium oxide. Therefore, if milled into a fine powder, this material might be employed as a supplemental cementitious material (SCM) or as a precursor for manufacturing geopolymers [42].

Many factors influence the reaction products produced during the alkaline activation of aluminosilicates, influencing the binder quality. Some of these parameters are the SiO_2_/Al_2_O_3_ ratio, the amount of reactive silica, the kind of alkali activator, and the reaction circumstances. By regulating these variables, optimal circumstances may be created to manufacture more significant quantities of a N-A-S-H gel. This material gives these systems their mechanical strength and endurance. During the alkali activation of aluminosilicate glasses, a N-A-S-H-type gel is produced as the primary reaction product, while zeolites are produced as secondary reaction products, much as it does with other aluminosilicate materials (such as fly ash). The composition of the products in this instance was conditioned by the glass composition, as opposed to other primary materials used to make alkaline cement, such as fly ash, where not all of the silica or alumina is reactive [43,44,45].

**Figure 4:**
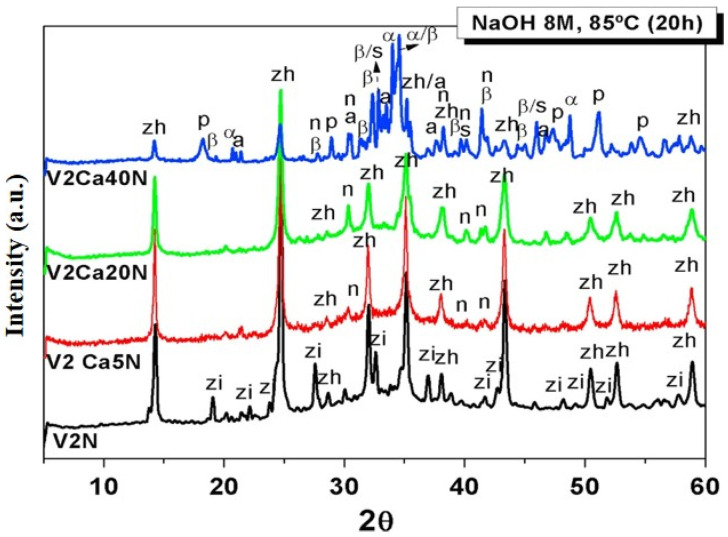
Diffractograms for 8M-NaOH-activated systems (V2N, V2CaN5, V2Ca20N, and V2Ca40N). Legend: zh = Na_8_(AlSiO_4_)_6_(OH)_2_·2H_2_O (42-0215); zi = Na_14_Al_12_Si_13_O_51_·6H_2_O, (28-1036); p = Ca (OH)_2_ (44-1481); a = NaAlO_2_ (33-1200); n**_c_** = Na_2_CO_3_ (37-0451); n = thermonatrite (08-0448); β = β-C_2_S (09-0351); α = Na_2_O·CaO·SiO_2_ (24-1069); s = Na_2_O·3CaO·2 SiO_2_ (23-0670) [46].

Garcia-Lodeiro et al. studied the alkali reactivity of aluminosilicate glass with a varied (from 0 to 40%) CaO content. The results show that the amount of CaO in the raw mix influences the growth of post-activation mechanical strength and the production of vitreous material. The diffractograms for all alkali-activated glass samples in this work showed a shift to higher values in the hump at 2*θ* values of 20 to 40° in anhydrous glass, a phenomenon often linked to N-A-S-H-type gel precipitation, as shown in Figure 4. The existence of this secondary reaction product was indicated by a set of lines connected to the crystalline zeolite phase hydroxysodalite. Furthermore, sodium carbonate-related diffraction lines were also seen. In the calcium-free system, a sodium aluminosilicate hydrate was found that was not seen in the other systems [46].

Using NaOH and KOH solutions as activators, Tashima et al. studied the characteristics and microstructure of alkali-activated glass fibre waste. After three days of curing at 65 °C, the compressive strengths of the mortar samples reached 77 MPa when activated with a 10 mol/L NaOH solution [47]. Furthermore, a study has shown that a NaOH solution works well in activating ground glass fibre (GGF). In this investigation, the GGF-based specimens demonstrated a high compressive strength of 80 MPa after 3 days, with curing at 60 °C for 24 h [48].

### 2.4. Lead Silicate Glass

Lead glass resembles soda-lime glass but contains more lead oxide than soda-lime glass. The average composition of lead glass is between 55% and 65% silicon dioxide, 18% to 38% lead oxide, and 13% to 15% sodium oxide or potassium oxide. Traditional applications for lead glass include cathode ray tubes (CRTs) and other electronic components [49,50]. Traditional cathode ray tubes (CRTs) are being phased out and destroyed en masse because of the fast development of electronics. The glass used in cathode ray tubes (CRTs) consists of three different parts—the panel glass (65%), the funnel glass (30%), and the neck glass (5%)—and is the most crucial part of the tube. CRT glass includes several harmful and hazardous chemicals, including lead; thus, it must be disposed of or recycled correctly as soon as possible. Lead glass is both a precious resource and a potential environmental saviour if appropriately recycled [51].

Using recycled lead glass as a viable alternative to natural aggregates in cement mortars or concrete blocks is a potentially attractive recycling solution. Smooth surfaces and minimal water absorption of the glass particles have been observed to improve the fresh characteristics of concrete when used instead of fine aggregates. The damping capabilities of cement mortar may be enhanced by the addition of CRT glass, particularly at low temperatures and at various vibration frequencies [52,53].

**Figure 5:**
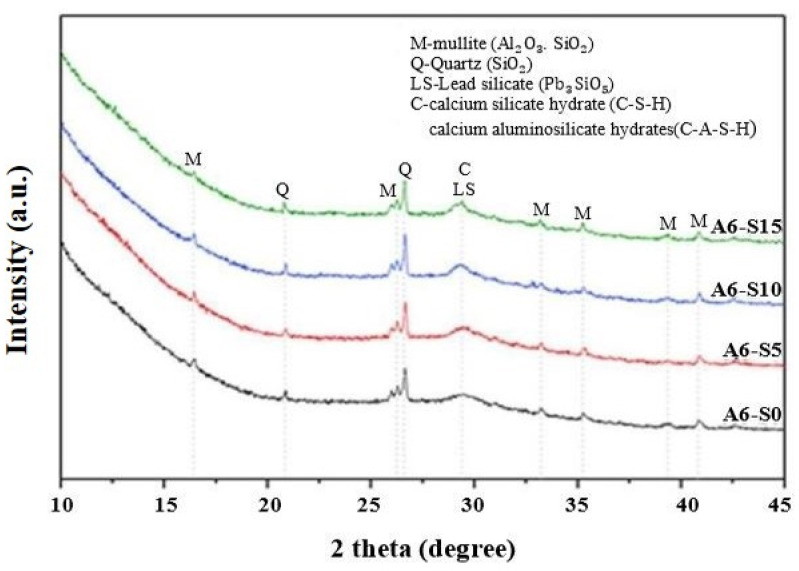
XRD patterns of geopolymer mortars with an alkali dosage = 6% [53].

In recent research, Long et al. [53] looked at recycling hazardous cathode-ray tube (CRT) glass by utilising fly ash–slag geopolymer mortar, a liquid water glass solution (${\mathrm{Na}}_{2}{\mathrm{SiO}}_{3}\;$ solution), solid sodium hydroxide (96% purity NaOH), and deionised water as an activator. Mullite and quartz comprised most of the geopolymer crystalline phase in the XRD investigation. Figure 5 illustrates the XRD patterns in which the C-S-H and C-A-S-H gels were also found. In general, the fly ash (FA) and the activator in the geopolymer gave the polymerisation process adequate silicate tetrahedrons. They broke down the aluminium tetrahedrons in the pore solution when there were enough silicate tetrahedrons. They bonded the calcium ions, forming gels that included C-A-S-H and C-S-H.

Meanwhile, some silicate and aluminium tetrahedrons were still present, which caused mullite to develop. Additionally, FA in the geopolymer released additional amounts of mullite. Moreover, the XRD patterns revealed the lead silicate crystalline form. Based on different microstructural test findings, the process of solidifying Pb ions of fly ash–slag geopolymer mortars incorporating CRT glass (FSGM-CRT) comprises physical encapsulation and chemical solidification via the production of ${\mathrm{Pb}}_{3}{\mathrm{SiO}}_{5}$. Moreover, raising the silica modulus enhances the geopolymer chemical solidification action on Pb ions [49].

### 2.5. Glass Wool and Rock Wool

Glass wool (GW) and rock wool (RW) are two common types of mineral wool with totally different chemical compositions. The primary components of regular stone wool are silicon dioxide (40–45 mass%), aluminium oxide (16–18 mass%), calcium oxide (16–18 mass%), and magnesium oxide (9–12 mass%), which is a very different composition to any other significant alkali-activated binder precursor. Its composition resembles typical soda-lime glass, with 60–65% of silicon dioxide, 16% of sodium oxide, and 7% of calcium oxide. Under alkali activation, the aforementioned oxides can promote the formation of binder gels like calcium-(sodium-) aluminate–silicate–hydrates (C-(N-)A-S-H), sodium–aluminate–silicate–hydrates (N-A-S-H), and layered double hydroxides (LDHs), which have been proven to give superior mechanical durability and strength [54,55].

**Figure 6:**
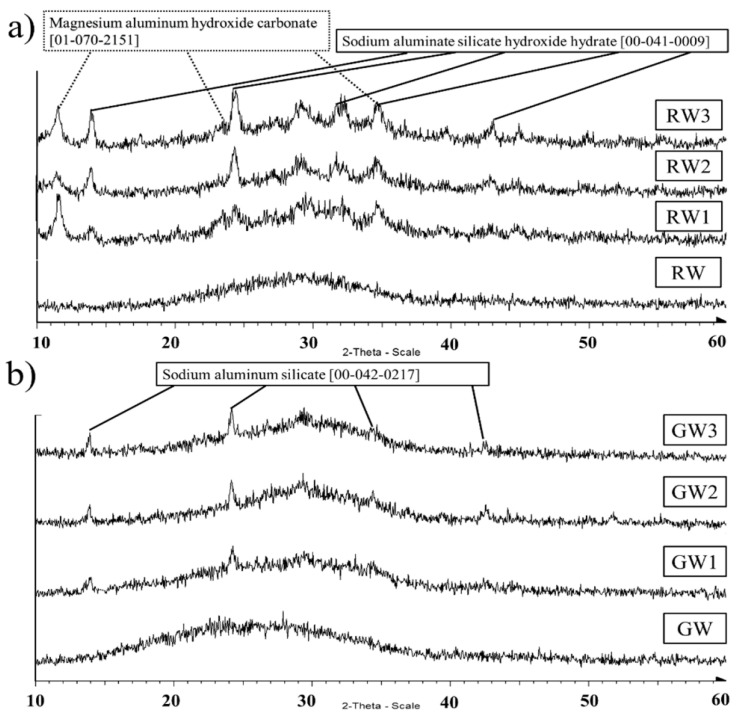
X-ray diffractograms of the (**a**) rock wool (RW); (**b**) glass wool (GW) and the alkali-activated samples [55].

Mineral wools are unique because their chemical composition and mineralogy are well suited for alkali activation. The fabrication of mineral wool and its use in building construction and demolition (C&D) led to the creation of mineral wool waste. There are presently 2.5 million tonnes produced annually in the European Union alone, and most of it is going to waste. The increased usage of mineral wool, a byproduct of the movement toward energy efficiency in building design, is predicted to increase global waste volumes [56]. Figure 6 shows the XRD pattern of rock wool and glass wool.

The fundamental variations in the chemistry of glass wool and stone wool are in the concentrations of Si, Ca, Al, Mg, and Na, which lead to a different kind of reaction pathway and binder gels. Earlier research revealed that $3{\mathrm{Na}}_{2}O.{\mathrm{Al}}_{2}O_{3}$-activated glass wool has both amorphous and crystalline N-A-S-H phases [55], while NaOH- and ${\mathrm{Na}}_{2}O.2{\mathrm{SiO}}_{2}$-activated glass wool binders comprise amorphous sodium silicate gels and a partly Ca-substituted N-(A-)S-H gel as an extra phase [54]. It is most likely due to the precipitation of amorphous sodium silicate gel and Ca-substituted N-(A-)S-H gels that the GW-NaOH, GW-${\mathrm{Na}}_{2}O.2{\mathrm{SiO}}_{2}$, and GW-${\mathrm{Na}}_{2}{\mathrm{CO}}_{3}$ samples have a fast setting time, high early strength, and high early-heat release [54].

Using mineral wool in alkali-activated materials has only been the subject of a few investigations [57,58]. In a recent investigation, the alkali activation technique was used to create mineral-wool-based mortars from a mixture of rock wool and glass wool. This study was carried out in three phases to elucidate the impacts of various factors on the mechanical strength, microstructural examination, and toxicity characteristic leaching technique. The findings showed that the mechanical strength, microstructural and mineralogical analyses, and leaching behaviour of heavy metals were all significantly influenced by the RW or GW content [59].

### 2.6. Volcanic Glass

Volcanic ash (VA) is an inexpensive and abundant substance studied as a potential replacement for ordinary Portland cement (OPC) and other sustainable alkali-activated building materials due to its chemical composition and amorphous atomic structure. A few steps exist to convert naturally occurring pozzolanic materials like volcanic ash (VA) into alkali-activated materials [60,61]. Unlike OPC materials produced by the clinkerisation process at 1350–1450 °C from many different natural resources, AAM materials are obtained at much lower temperatures of 25–100 °C or 600–700 °C if the calcination procedure is necessary. Consequently, manufacturing ordinary Portland cement (OPC) using natural pozzolanic materials significantly reduces carbon dioxide emissions [62]. In addition, there are vast untapped reserves of natural pozzolanic minerals throughout the planet [63].

There are two classes of VAs concerning their alkaline activation. The first category includes geopolymers and inorganic alkaline polymers, and both may be defined as alkali-activated binders without OPC. In this class of reactions, zeolitic precipitates and sodium or potassium aluminosilicate hydrate (N-A-S-H) gels are the primary end products [64,65]. A second, less-researched category is mixed cement, which combines OPC with alkali-activated aluminosilicate binders. The aluminosilicate precursor, VA, makes up the bulk of the mixed cement, whereas OPC makes up a tiny percentage (30%). The primary byproducts of the process are highly intricate cementitious gels. Aluminosilicate hydrate (C -(N-) A-S-H) gels consist of sodium and calcium in 10M NaOH (NH) and other very alkaline media. The calcium silicate hydrate (C-S-H) product predominates in mildly alkaline conditions, such as 2 M NH [66,67].

**Figure 7:**
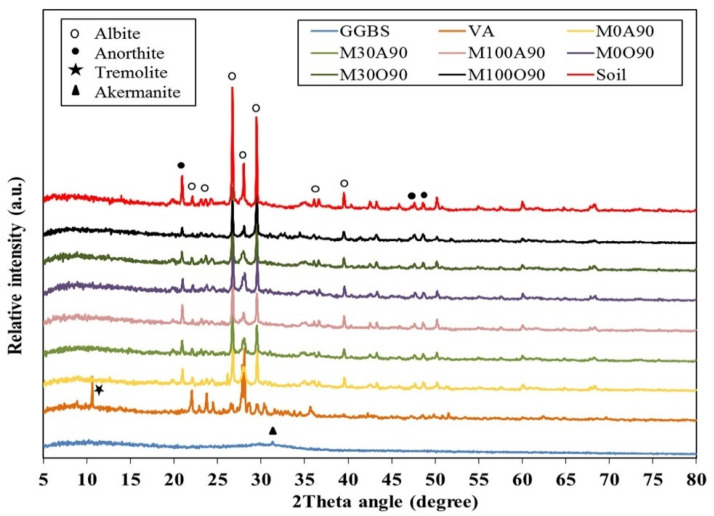
XRD patterns of the binders, untreated soil, and stabilised samples [68].

In recent work, Bernardo et al. created inorganic polymers from a mixture of soda-lime glass and volcanic ash using low molarity NaOH (3 M) activation. Through direct foaming, very porous glass ceramics were produced. In this study, zeolite-like gels are produced by curing a combination of volcanic ash and glass waste at nearly room temperature, primarily in the form of Na-based zeolites such as zeolite NaP and hydrocancrinite [60]. Miraki et al. explored the possibility of using volcanic ash (VA) and alkali-activated ground granulated blast-furnace slag (GGBS) as green binders. The XRD patterns showed that adding GGBS reduced the intensity of the crystalline phase peaks and increased the amount of amorphous phase, as shown in Figure 7. However, altering the curing condition does not impact the geopolymer crystalline components, proving that the observed variations in strength values are caused by changes in the amorphous phase [68].

### 2.7. Waste-Derived Glasses

Most hazardous inorganic waste comes from industry. However, it may also originate from the destruction of buildings and public infrastructure and combustion operations, such as municipal solid trash incineration. From disposal in a landfill to recovery, all management strategies involve some kind of stabilisation step that is achieved through a chemical or physical process; vitrification, which can be used on even radioactive waste, is arguably the most effective, but it is also costly and resource-intensive [69].

The second thermal treatment that waste-derived glasses typically undergo when being transformed into new construction materials may compromise sustainability, despite several optimisations. This treatment involves replacing the lengthy nucleation and crystal growth treatments typically performed on bulk glass pieces with the faster sinter–crystallisation of waste-glass powders. Consequently, the possibility of a “cold” transformation, characteristic of alkali-activated materials, is appealing as a way of lowering transformation costs [70,71].

**Table 1:** Glass types and their composition.

Glass Type	Applications	Approximate Composition	Description	Ref.
Soda-lime	Container glass Window panes Building sector	$70\%\;\mathrm{Si}O_{2}$ $11\%\;Na_{2}$O 11% CaO 1% MgO $2\%\;Al_{2}O_{3}$	Prepared by heating a mixture that includes limestone ($\mathrm{CaC}O_{3}$), silica sand ($\mathrm{Si}O_{2}$), soda ash ($Na_{2}$$CO_{3}$), and a few additional ingredients. Crushed jars, bottles, containers, discarded windows and doors, and scraps from glass unit production are typical sources of soda-lime glass waste.	[33]
Borosilicate	Pharmaceutical glass Laboratory glassware Optical glass	70–80% $\mathrm{Si}O_{2}$ $13\%\;B_{2}O_{3}$ $4\%\;Na_{2}$O $2\%\;Al_{2}O_{3}$	Typically made up of silicon dioxide, boron trioxide, aluminium oxide, and a few alkaline earth oxides. Due to its low alkali content, this glass offers excellent chemical durability and thermal shock resistance. It finds widespread use as laboratory equipment and pharmaceutical storage containers in the chemistry sector.	[72]
Aluminosilicate	Fibre glass Mobile phone screens Combustion tubes	$56\%\;\mathrm{Si}O_{2}$ 23% CaO $13\%Al_{2}O_{3}$ $5\%{\;B}_{2}O_{3}$	Common uses include insulation, fibre optic cables, strengthened parts, and smartphone displays.	[73]
Lead silicate	TV screens (CRT) Artistic ware Absorption of X-rays	55–65% $\mathrm{Si}O_{2}$ 25–30% PbO 12–16% $Na_{2}$O 13–15% $K_{2}$O	Often just called “crystal” for short. It is used often in ceramic glazes, cathode ray tubes (CRTs), and other artistic glassware. PbO is present in large quantities, which gives the product its shiny appearance.	[74]
Glass wool	Pipe insulation Suspended ceilings Higher-temperature insulation	$63\%\;\mathrm{Si}O_{2}$ 8% CaO $16\%\;Na_{2}$O 3% MgO 1–2% $Al_{2}O_{3}$	Construction and demolition (C&D) waste and mineral wool production are two major sources of glass wool waste. The majority of this mineral waste is unutilised at the moment.	[75]
Volcanic glass	-	$46.8\%\;\mathrm{Si}O_{2}$ $16\%\;Al_{2}O_{3}$ $11.9\%\;{\mathrm{Fe}}_{2}O_{3}$ 9.8% CaO 6.3% MgO $2.8\%\;Na_{2}$O $1.8\%\;K_{2}$O	Easily accessible and inexpensive, volcanic ash (VA) is a substance with many potential applications. Due of its chemical composition and amorphous atomic structure, volcanic ash has been proposed as a replacement for Portland cement and other alkali-activated building materials.	[76]

**Figure 8:**
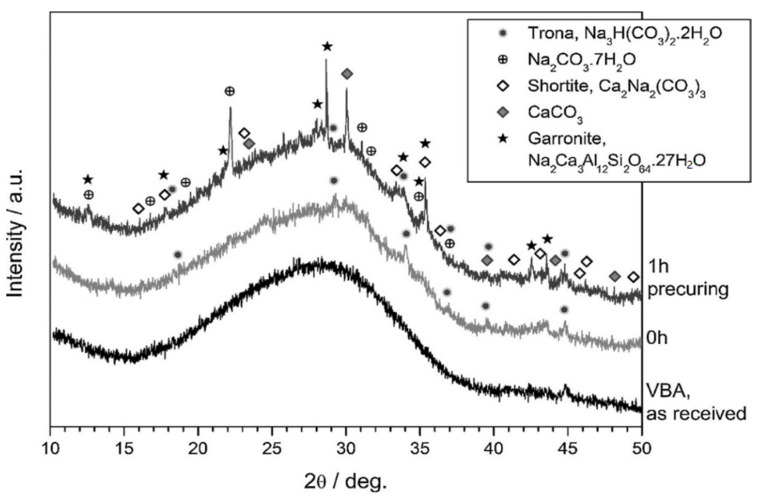
Mineralogical analysis of VBA, in the as-received condition and after activation (pre-curing time of 0–1 h) [4].

Rincon et al. [4] produced extremely porous glass–ceramics from vitrified municipal solid-waste incineration (MSWI) bottom ash using the inorganic gel casting process. Bottom-ash-derived glass suspensions experienced a gradual hardening in this experiment at low alkali molarity and over a short period. Due to a significant crystallisation, the fire had little effect on the open-celled structure that had emerged during low-temperature foaming. Patterns from the high-resolution X-ray diffraction (Figure 8) investigation indicate that vitrified bottom ash (VBA) glass was prone to the production of a genuinely geopolymeric-like (zeolitic) gel, amorphous (with no pre-curing) or semicrystalline (with pre-curing) [4].

## 3. Alkali Activation Mechanism in Waste Glass

**Figure 9:**
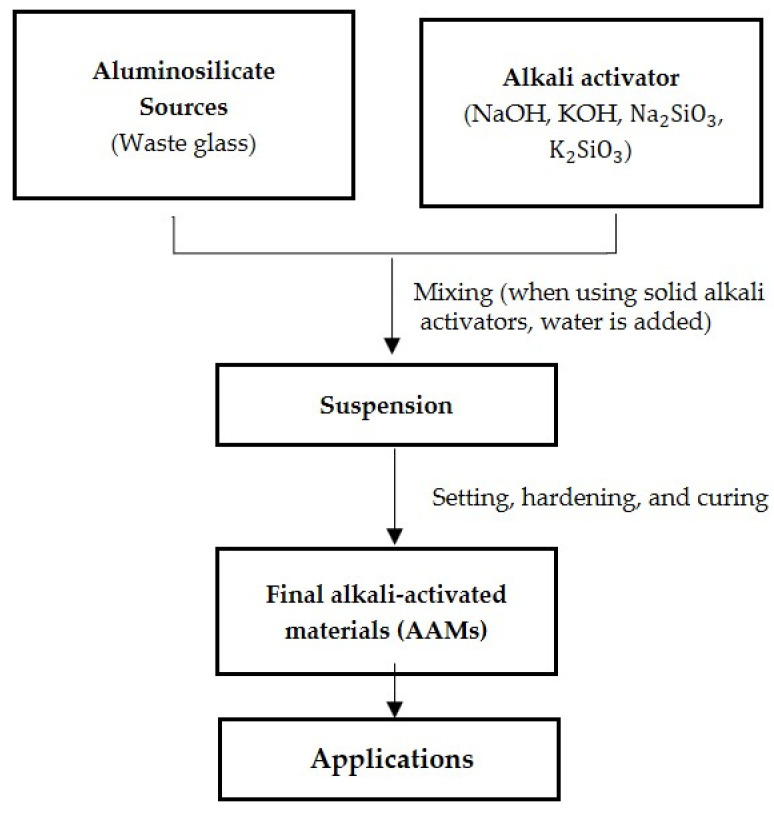
Overview of alkali-activated material synthesis.

Alkali activation is a chemical process that converts partially or entirely amorphous vitreous materials into cementitious composites [76]. The process needs an alkaline medium to dissolve a particular quantity of silica and alumina to promote the surface hydrolysis of the raw material particles. Alkaline activators, which can be either single or mixed solutions, produce such a medium [77]. The most common alkaline solution or alkali-activator type is a hydroxide solution, such as sodium hydroxide (NaOH) or potassium hydroxide (KOH). Sodium hydroxide or potassium hydroxide are mixed with sodium metasilicate (${\mathrm{Na}}_{2}{\mathrm{SiO}}_{3}$) or potassium metasilicate ($K_{2}{\mathrm{SiO}}_{3}$) to form the respective alkaline solutions [78,79]. Figure 9 represents the method for producing alkali-activated materials.

The gelation mechanism and reaction products are chemically dependent on waste glass composition. The amorphous, silica-rich glass powder dissolves when treated with an alkaline activator [80,81]. Due to the presence of amorphous SiO_2_, glass exhibits pozzolanic activity and creates a silicate network [82,83]. Initially, raw glass foam forms an inorganic C-S-H gel with silica (${\mathrm{SiO}}_{2}$) and calcium-oxide (CaO) in the waste glass. However, in the early stages of curing, this reaction is sluggish. Additives are advised to play a crucial role in finishing the hydration processes or creating polymeric bonds due to the delayed bond development within waste glass particles. To hasten the hydration of waste glass particles, a CaO source such as slag is needed to enhance the calcium silicate hydrate gel production and to improve the raw glass-foam stability. On the other hand, a supply of ${\mathrm{Al}}_{2}O_{3}$ is advised for the alkali activation procedure to accomplish the geopolymerisation of waste glass particles. Figure 9 represents the method for producing AAMs [34].

Raw glass foam forms Si-O-Si networks with a medium Si/Al ratio, while raw glass foam with a low Si/Al ratio forms Si-O-Al and Al-O-Al networks [84,85]. Some glasses, such as boro-aluminosilicate glasses, are alumina-rich and have negligible CaO, and their alkali activation leads toward the production of a hydrated N-A-S-H gel [46,86]. The stabilisation of the growing network inside the matrix can be achieved by adding waste-derived additives, such as alumina-rich fly ash [87] and remnants from the plasma processing of urban waste [88], which positively affects the pore structure of glass foam. These solid silicate structures help stabilise the pores in the raw glass. The growth of these networks relies on the $\mathrm{Si}O_{2}$, CaO or ${\mathrm{Al}}_{2}O_{3}$ dissolving from the raw materials, which increases with particle fineness and depends on the activator’s characteristics and the activation process [89]. A recent study on boro-aluminosilicate glass aimed to decipher the mechanism of hardening at low molarities and to promote the hydration of glass surfaces, with reduced dissolution (i.e., the release of glass components in solution). Particles may connect by the condensation of SiOH groups and the formation of Si-O-Si bonds across adjacent particles [23]. Table 2 provides an overview of alkali activators used in previous studies.

### 3.1. Concentration of Alkaline Solutions

In AAM research, an alkaline solution is applied to break down the raw materials needed to create the monomers for the alkali activation [91]. Alkali cations (${\mathrm{Na}}^{+}$/$K^{+}$) and an anion of hydroxides make up an alkaline solution (MOH, where M = ${\mathrm{Na}}^{+}$ or $K^{+}$) (OH– ions). The negative charge of the aluminate tetrahedral ${\mathrm{AlO}}_{4}$ is stabilised by the presence of both cations and anions in an alkaline solution, which permits the breakdown of bonds on the surface of the raw materials [92,93]. A highly concentrated alkaline solution is needed to achieve the high $\mathrm{Si}O_{2}$ dissolving rate from waste and the early gelation with minimal curing time [94].

However, excessively alkaline environments may also unnecessarily delay the breakdown of aluminosilicate compounds and polymerisation due to low ion mobility [95]. Moreover, solidifying concentrated activators might result in a greater number of large and irregular structures [96,97]. Thus, to maintain pore homogeneity and sustainability, Rincon et al. [4,34] suggest using an activator with a lower molar concentration, ranging from 2–4 M, and an activator to solid ratio of 0.45 for the production of glass-based alkali-activated materials. Depending on the nature of the activator and the mixture percentage, the appropriate concentration range for the alkali activator is 2–8 M [22,33,98]. For the alkali activation of waste glass, NaOH, KOH, and ${\mathrm{Na}}_{2}{\mathrm{SiO}}_{3}$ are frequently used alkali activators [82,99,100]. CaO and $\mathrm{Ca}{\left(\mathrm{OH}\right)}_{2}$ are alternative sustainable activators that may be employed to speed up hydration; these activators are currently present in geopolymer foams [101,102]. The activation period and pre-curing conditions, along with the activator concentration and type, may also affect the characteristics of the final product.

### 3.2. Pre-Curing Time

**Figure 10:**
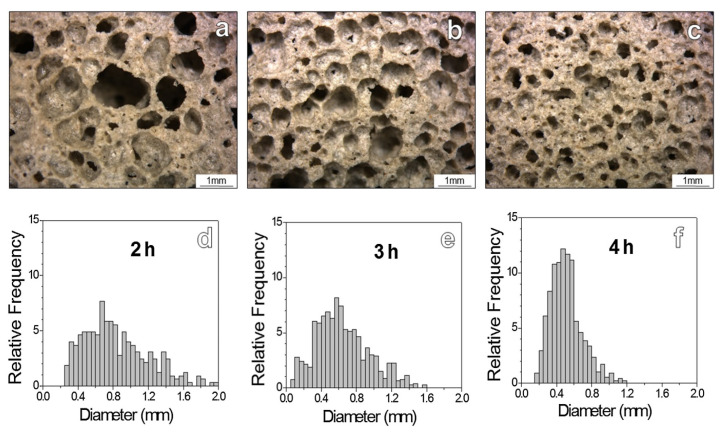
Hardened foamed gel microstructures (**a**–**c**) and their pore size distribution (**d**–**f**). The foams after 2 h exhibited a quite coarse microstructure, with many big interconnected pores surrounded by smaller ones, as an effect of coalescence between adjacent bubbles (**a**). The more pronounced pseudoplasticity with a longer curing step progressively reduced the coalescence (**b**,**c**); in particular, a curing step of 4 h was found to enhance the uniformity of foams (**c**) [34].

Silica dissolves more readily with extended activation and pre-curing times, while gelation increases. To achieve the necessary ${\mathrm{SiO}}_{2}$ dissolution and gelation in a waste glass mix, Rincon et al. [22,34,87] advises 3 h of alkali activation and 4 h of pre-curing (75 °C). The solution becomes more viscous as gelation progresses, which improves pore dispersion. As shown in Figure 10 [78], the glass-foam pore size decreases as the pre-curing time rises, and its pore distribution becomes more homogeneous. Geopolymer and alkali-activated cement foams are being created using a similar technique with a much shorter mixing time and no pre-curing step [97,100,103].

Therefore, the processing time could be optimised to produce the desired viscosity and reactivity required for homogeneous pore distribution by adding comparable geopolymer and cement foam additives. The right foaming chemicals and procedures should be used for a uniform pore distribution.

## 4. Properties of AAMs Incorporating Glass Waste as a Raw Material

### 4.1. Workability

The integration and increased amount of GP improves the workability of alkali-activated materials, which is attributed to the glass powder’s physical characteristics. Compared with metakaolin, glass powder has a smaller surface area and larger particle size, which reduces the need for the liquid to wet the precursor and increases the amount of water available for the mixture workability. Si et al. used the mini-slump test to assess the workability of alkali-activated materials (AAMs) manufactured using up to 20% glass powder as a partial substitute for metakaolin [104,105]. The research results showed that the workability of alkali-activated materials was enhanced with a higher concentration of glass powder.

**Figure 11:**
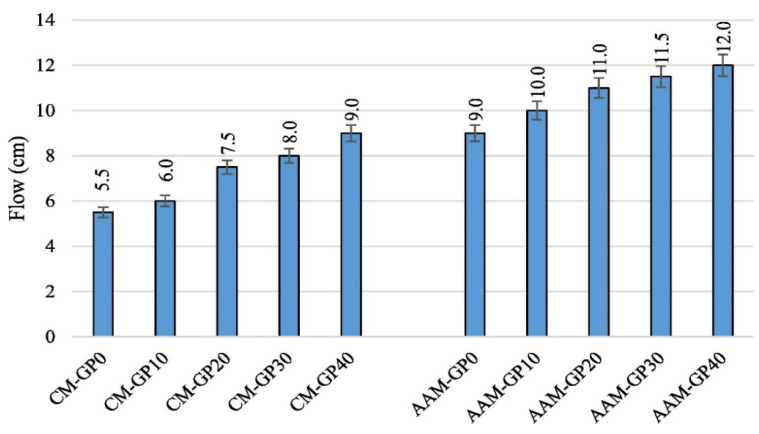
Flow of the fresh mortar with different glass powder (GP) concentrations. CM = control mix [108].

In another investigation, Jiang et al. replaced up to 30% of the FA with glass powder in AAMs. The outcomes from this study also showed an improvement in the fresh AAMs’ workability at higher glass powder concentrations. Compared with AAMs produced using just FA as the precursor, the flow increased by 3%, 11%, and 14%, respectively, when glass powder was used as a 10%, 20%, and 30% replacement for FA. The flat surface of glass powder, which imbued it with a lower water absorption ability, was thought to be responsible for the increased flow in fresh AAMs [106]. These findings are consistent with those of Samarakoon et al., who found that using GP as a precursor instead of up to 30% FA improved the workability of AAMs by up to 80% [31]. These findings concur with those of Lu and Poon [27], who found that adding glass to AAMs instead of 30% FA and slag (SL) increased their workability. A similar finding was also made by Dadsetan et al., who conducted a thorough analysis of the impact of glass powder on the rheological characteristics of AAMs based on metakaolin [107]. Similar findings were made, as shown in Figure 11, by Shoaei et al. [108], where up to 40% glass powder (GP) was utilised as a precursor in AAMs instead of SL. Comparing the AAM created with solely SL as the predecessor to the AAM made with GP as a replacement for 10%, 20%, 30%, and 40% of SL, the flow was increased by 11%, 22%, 28%, and 33%, respectively [108].

### 4.2. Setting Time

**Figure 12:**
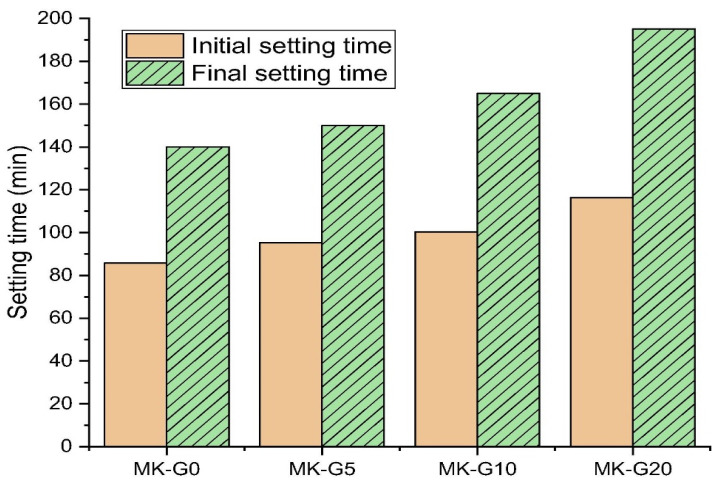
Setting times for various glass powder contents in geopolymer mortars based on metakaolin [104].

Based on the results from Si et al.’s evaluation of setting time using the Vicat needle technique, Figure 12 shows that the AAMs’ setting periods were delayed due to the higher glass content employed to partially replace metakaolin (MK) as a precursor. The increase in setting time, based on the addition of waste glass, was explained by a decrease in reaction rate that led to a slower polycondensation rate and a later emergence of activation products [109]. This finding aligns with that of Liang et al., who replaced up to 30% of MK with waste glass. Samarakoon et al. had a similar discovery, reporting that using glass powder as a precursor instead of up to 30% FA caused an increase in both the initial and ultimate set durations. However, the physical characteristics of the GP were responsible for the longer setting time that occurred from the integration of GP because they made more water accessible in the fresh AAM [31,110]. Additionally, Huseien et al. showed that the set durations were extended when nano glass was utilised as an up to 20% substitution for the SL in alkali-activated materials [111].

In contrast, the research by Jiang et al. showed a reduction in the final setting time of the AAMs when GP was employed as a substitute for up to 30% FA. Compared with the AAM created with just FA as the precursor, the final setting time was reduced by 6.8%, 21.9%, and 26.6%, respectively, when FA was replaced with 10%, 20%, and 30% GP as a precursor [106]. The more reactive silica found in GP contributed to the GP’s ability to build three-dimensional alumina–silicate structures more quickly, which reduced the setting time for GP integration. Similar results have been reported by Liu et al., who found that using GP to replace 40% of the FA reduced the setting time [112]. However, similar to AAMs manufactured with other precursors, the setting durations involving GP may be sped up by heat curing for the first 24 h after casting [113].

### 4.3. Density

**Figure 13:**
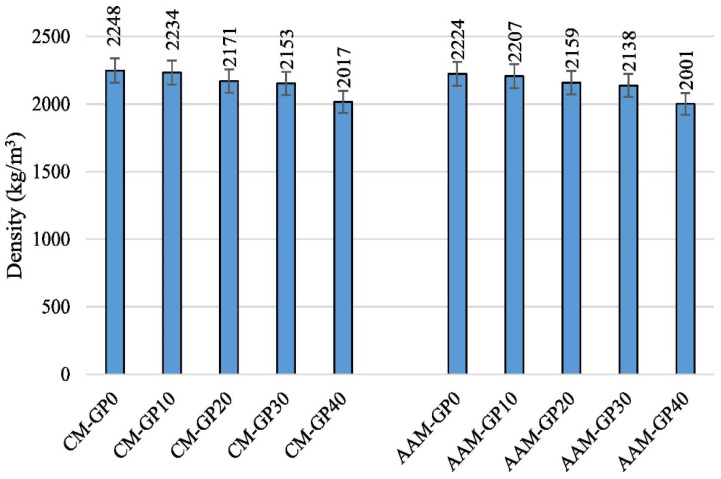
Influence of glass powder concentration on density [108].

It has been discovered that the density decreases when GP is used as a precursor in AAMs. According to the research by Shoaei et al., using GP as a precursor instead of SL up to 40% of the time causes a 10% loss in density, as shown in Figure 13 [108]. Because GP has a lower specific gravity than other precursors like SL, the density of the AAMs decreases when GP is used. According to research by Liang et al., AAMs containing GP as a 10% or 20% substitute for MK had greater densities than those without GP [110]. However, the density of the AAMs decreased when the GP content was raised to 30%. AAMs created with GP as a 10% or 20% substitute for MK had a greater density. This was associated with the generation of more reaction products, which led to a higher microstructure density.

### 4.4. Compressive Strength

**Figure 14:**
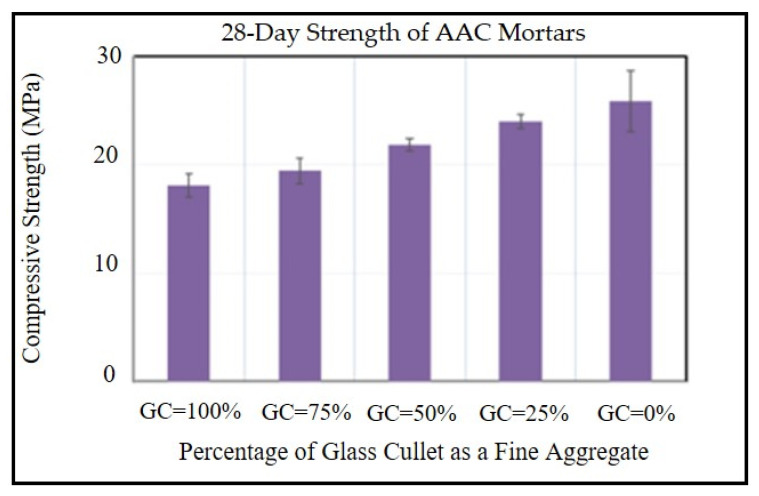
Compressive strength of AAC mortars made with varying recycled glass cullet concentrations [21].

Regardless of activator type, it has been discovered that using GP as a substitute for SL by up to 100% reduces the compressive strength of AAMs [114]. This finding is in line with that of Zhang et al., who discovered that using GP as a precursor in place of SL either partially or completely resulted in reduced strength, as seen in Figure 14 [21]. The poor reactivity of GP as compared to SL leads to a reduction in the compressive strength of AAMs with higher GP content. Similarly, Tho-In et al.’s work showed that the loss in compressive strength was caused by the integration of two kinds of GPs as a partial replacement for FA as a precursor. The compressive strength of AAMs made with GP as a 10%, 20%, 30%, and 40% replacement for FA was, respectively, 10.1%, 10.9%, 13.3%, and 27.1% lower in comparison with AAMs manufactured with just FA as the precursor. The rise in the silicate-to-aluminate ratio, which led to the development of low-crosslinked products, was responsible for the decrease in compressive strength [115,116].

**Figure 15:**
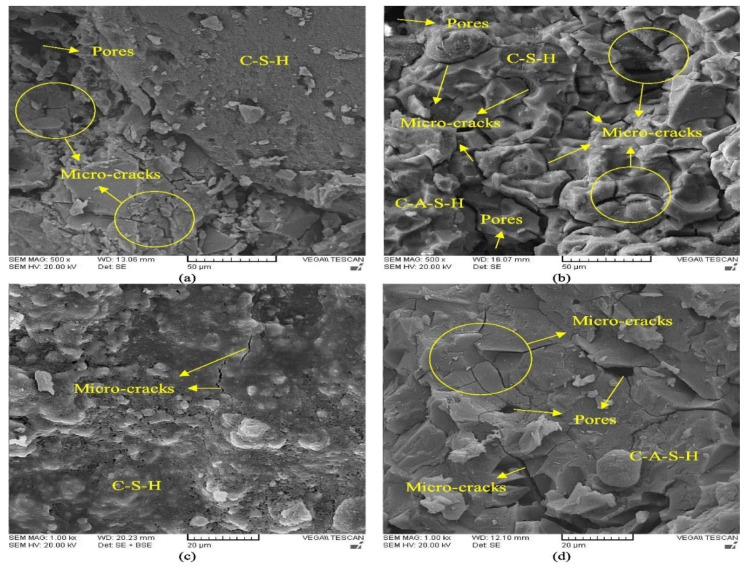
SEM images of (**a**) CM-GP0, (**b**) AAM-GP0, (**c**) CM-GP20, and (**d**) AAM-GP30 [108].

These findings concur with those of Xiao et al., who discovered that compressive strength was decreased when GP was used to substitute up to 100% of the FA. Additionally, it was found that using GP to replace up to 100% of the SL resulted in reduced compressive strength. GP’s weaker reactivity than SL leads to less product formation and a more porous structure, so the compressive strength decreased when GP was added [25,78]. According to Xiao and Shoaei et al., the AAM microstructure becomes more porous as its GP content increases, as seen in Figure 15 [25,108]. Contrarily, Redden and Neitthalath showed that using GP to replace 50–100% of FA increased the compressive strength for various starting curing temperatures [117]. Similarly, Novais et al.’s research found that adding 12.5% GP instead of MK increased the compressive strength by 31.4%. These contradictory observations may result from greater curing and from using only sodium hydroxide as an activator [109].

## 5. Applications of Waste-Glass-Based Alkali-Activated Materials

### 5.1. Soil Stabilisation

The design and construction of infrastructure structures are subject to geotechnical restrictions when built on soft soils. For a long time, engineers have turned to soil stabilisation as a way to fortify flimsy soft soils. The two primary types of stabilisation methods are chemical and mechanical. The soft soil is first mixed with a chemically active ingredient such as OPC, lime, or, more recently, geopolymer and AAMs and then mechanically compacted. Hence, pozzolanic interactions between soil particles and binders enhance the soil quality. Soil enhancement methods using unique and environmentally friendly materials like geopolymers and AAMs based on WG have been developed recently [118,119,120,121].

Soil stabilisation using geopolymers based on WG alone has been investigated [122], even though WG is sometimes employed with a typical binder like OPC. It has been reported that the initial synthesis temperature of the alkali activator can affect the rate of dissolution of silica and alumina from the base materials (WG and soil). The effective curing temperature range lies between 25 °C and 80 °C and has a vital role in accelerating the AAM gel formation, leading to increased final resistivity in stabilised soils [123].

Grubb et al. [124] concluded that using glass dust to stabilise soils made of degraded materials significantly improved their properties by lowering their water content, organic matter, and plasticity index while being much more cost- and environmentally friendly than when using Portland cement or other techniques. The favourable disposition of cementitious materials formed with glass powder for soil stabilisation was made evident in experiments conducted by Garca del Toro et al. [125]. Their research showed that the gels created by glass powder during cementitious material setting had the unique ability to self-heal the soils.

### 5.2. Road-Base Stabilisation and Tile Production

Xiao et al. used GP-based AAMs to stabilise road bases, comparable to soil stabilisation. As a precursor to creating AAMs, a stabilising substance for road bases, the GP was taken from glass containers and combined with FA. The study findings demonstrated that the GP and FA had more excellent amorphous silica contents than the GP alone and that this combination improved the strength performance of road bases [126].

**Figure 16:**
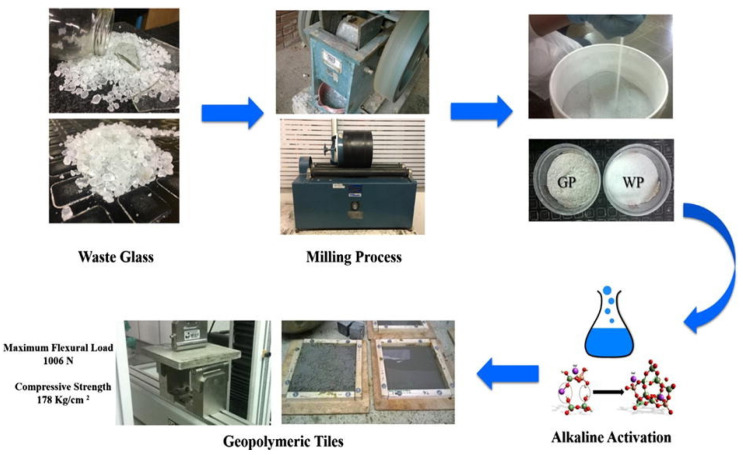
The fabrication process for waste-glass-based alkali-activated tiles [90].

In order to create AAM-based tiles, Rivera et al. [90] used GP derived from soda-lime window glass, fluorescent bulbs, and glass bottles. According to their research results, creating titles with high mechanical performance is possible using simply GP as the aluminosilicate precursor. When the GP was utilised as a precursor, a flexural load capacity of up to 1006 N was attained. The fabrication procedure and flexural load analysis of the GP AAM-based titles are shown in Figure 16.

### 5.3. Geopolymer Concrete

Geopolymer concrete is an alternative cementitious material that is generally expressed as alkali-activated binders. It was developed by the polymerisation of different aluminosilicate precursor materials in the presence of an alkaline activator solution, which led to the formation of the calcium aluminate silicate hydrate gel (C-A-S-H) and sodium aluminate silicate gel (N-A-S-H) as final reaction products [127]. Compared with OPC-based concrete, geopolymer binders have higher mechanical properties, fire resistance [128] and acid resistance [129], and lower thermal conductivity due to the high proportion of aluminosilicate source material [130]. Materials with a high silica content, like waste-glass powder (WGP), can be used as a cementitious additive in producing environmentally friendly geopolymer concrete. There are several negative environmental effects caused by waste glass, but using WGP in geopolymer synthesis and characterisation could provide a viable alternative [116,117]. These benefits include (1) preserving raw resources from OPC manufacturing, (2) drastically reducing carbon dioxide and other greenhouse emissions, (3) recycling trash and industrial by-products into valuable products, and (4) saving a lot of money and energy.

Samarakoon et al. studied the influence of soda-lime glass powder as a substitute for FA on the rheology and microstructural characterisation of geopolymer binders. The 30% WGP replacement improved the workability, setting time, and microstructural properties in the experiments [31]. Another study found that the compressive strength of a GGBS/FA-based geopolymer system increased by 35% after 28 days when WGP was used as a 30% substitute. Further microstructural studies demonstrated a more robust polymerisation process, with C-A-S-H and N-A-S-H gels as the final products [131].

### 5.4. Thermal and Acoustic Insulation

Glass foams made from waste glass (WG) are an environmentally benign, long-lasting, and high-performance material that can be used to insulate buildings from heat [34]. Glass foam is a porous, light-weight composite material with foam that has a very low heat conductivity and a suitable level of fire resistance and acoustic insulation [132,133]. It is a sustainable insulating material that may be entirely produced from used glass bottles that have been recycled [134].

The open-cell and closed-cell forms of pore morphology in glass foams can be categorised. The possible application of glass foams largely relies on the ratio of open and closed porosity [135]. The existence of micro, round, and closed cells in porous materials is an efficient way to create high thermal insulation and high coupling strength, according to the concept of heat transmission [136,137,138]. Rincón et al. produced glass ceramic foams by applying “weak alkali activation” to aqueous suspensions made of soda-lime glass and coal fly ash. High porosity, low thermal conductivity, and adequate mechanical strength make the developed glass–ceramic foams suitable for use as thermal and acoustic insulation building materials [86]. Ramteke et al. manufactured glass foams by weak-alkali activation, gel casting, and low-temperature sintering using fibre and opal waste glass. Glass-fibre-based foams showed a good strength-to-density ratio. Opal-glass-based foams displayed excellent, consistent open porosity [22].

Tameni et al. upcycled pharmaceutical glass waste to create porous ceramics. The partial dissolution of boro-aluminosilicate glass fine powders in a weak alkaline solution of sodium and potassium hydroxides, known as alkali activation, is an environmentally friendly method for recycling glass. Face-brick-like products may be produced immediately after cold consolidation or with low-temperature (700 °C) firing, depending on the composition [38]. Moreover, specific formulations produce highly porous glass foams that may be employed for acoustic and thermal insulation.

### 5.5. Other Applications

Porous glass foams were explored for their filtration capabilities in eliminating undesirable minerals from brackish water for industrial salt production [139]. Many industrial applications exist for the glass microspheres created by alkali activation, such as using hollow glass microspheres as a secure method for hydrogen storage [73,140]. In recent work, fibreglass waste (FGW) was alkali activated in an aqueous solution at various sodium/potassium hydroxide concentrations. The materials were fed into a methane–oxygen flame that was around 1600 °C in temperature. X-ray diffraction studies confirmed the creation of various hydrated compounds; these compounds broke down during flame synthesis to produce porous glass microspheres (PGMs). The use of highly concentrated activating alkaline solutions was found to enhance pore development. The activation with a 9 M KOH aqueous solution resulted in the highest homogeneity and yield of PGMs. The produced PGMs could be used in applications for hydrogen storage or as filtration materials [73].

## 6. Conclusions

Recycling aluminosilicate industrial wastes, like glass cullet, as precursor materials for the production of AAMs, is a crucial step toward sustainable waste management because of its numerous environmental advantages, such as relieving the burden of landfilling costs and environmental effects, the potential to reduce energy consumption and CO_2_ emissions compared with the traditional Portland cement technology, the preservation of natural resources for binder production, and the efficient immobilisation of heavy metals found in glass debris. When compared to concrete waste and other forms of construction and demolition waste, glass waste offers ideal synthesis conditions for alkali activation due to its more reactive SiO_2_ content. This study has reviewed how waste glasses are used to produce AAMs, which leads to the following conclusion:

- Waste glasses from various sources may be used to produce AAMs. The reactivity of glasses was significantly impacted by the various oxide amounts in waste glasses and, subsequently, the performance of alkali-activated materials and hydration products.
- Using different types of glass results in different hydration products for AAMs. Alkali-activated low-calcium waste glass precursors mainly produce NA-S-(H) gels, while alkali-activated high calcium materials primarily produce C-(A)-S-H gels.
- Similar levels of success have been seen with the utilisation of processed glass waste in foam glass as with its denser counterpart. Glass cullet is a valuable resource due to its low weight, excellent thermal and chemical durability, and solid insulating qualities, which may be utilised in a range of applications. However, the foaming process’s efficiency also relies on several intrinsic characteristics of the glass employed, including the kind of glass, its fineness, and its reactivity, in addition to the foaming agent and sintering temperatures and timeframes.
- Lightweight inorganic polymers may be produced by activating NaOH solutions with low molarities; the ideal concentration range for the alkali activator is between 2 and 8 M, depending on the kind of activator and the mixture percentage. NaOH and KOH are the alkali activators often used for the alkali activation of waste glass. The activation duration, pre-curing conditions, activator concentration, and activator type may also influence the features of the finished product.
- With applications ranging from construction to fireproof safety, carrier media in bioreactors, adsorbents, energy storage, medications, the solidification/stabilisation of contaminants, pH buffers, and radioactive waste containers, alkali-activated materials (AAM) have a lot of potential as versatile materials.

Every year, there is an increase in the amount of glass waste produced throughout the globe. At the same time, there is an increase in building activity, which puts an even more tremendous strain on the environment. However, the building industry may absorb a significant amount of waste in the form of alkali-activated materials. Reusing waste glass in the manufacturing of AAMs would have several advantages, including reduced greenhouse gas emissions, extended landfill life, significant energy savings, and environmental protection.

## Figures and Tables

**Table 2:** Overview of alkali activators and the concentrations used for waste glass.

Type of Waste Glass	Curing Time (h)/Temperature (°C)	Alkaline Activator	Activator Concentration (mol)	Major Findings	Ref
Glass bottles, window glass, fluorescent lamps	24/70	NaOH	4, 6, 8, 10	The ideal value for the activator (4–6 M), but as materials age, they lose strength due to phase shifts, breaking, and shrinkage. A C-S-H type gel was the primary reaction product.	[90]
Mixed-colour soda-lime glass	20/85	$\mathrm{NaOH},\;\mathrm{NaOH}/{\mathrm{Na}}_{2}{\mathrm{SiO}}_{3}$, KOH	-	A Si-high, Al-low gel was the primary reaction product, regardless of the nature of the activator or curing method. When alkaline-activator concentrations are high, less porous and more robust pastes with more compact microstructures are produced.	[33]
Glass wool, stone wool	72/40	$\mathrm{NaOH},\;\mathrm{NaOH}/{\mathrm{Na}}_{2}{\mathrm{SiO}}_{3}$	5	The outcomes show that both precursors are appropriate for the alkali activation procedure. When sodium silicate or a solution of sodium silicate and NaOH was utilised, the compressive strength after three days of curing at 40 °C was superior in glass wool compared with stone wool.	[88]
Window glass, hollow glass, windshield glass	24/60	KOH	1, 2, 3, 5, 7, and 10	The concentration of the activating solution and the time of the curing process at 60 °C both affect compressive strength. Regardless of the type of glass, 3 mol/L of activating solution (KOH) is the ideal concentration.	[20]
Pharmaceutical boro-aluminosilicate glass	-/40	NaOH/KOH	2.5	When making alkaline solutions, industrial mud can be used in place of water. Products similar to facing bricks can be produced directly by cold consolidation or following the application of low-temperature (700 °C) firing, depending on the formulation.	[38]

## Data Availability

The data presented in this study are available upon request from the corresponding author.
